# Intracranial AAV‐IFN‐β gene therapy eliminates invasive xenograft glioblastoma and improves survival in orthotopic syngeneic murine model

**DOI:** 10.1002/1878-0261.12020

**Published:** 2017-01-18

**Authors:** Dwijit GuhaSarkar, James Neiswender, Qin Su, Guangping Gao, Miguel Sena‐Esteves

**Affiliations:** ^1^ Horae Gene Therapy Center University of Massachusetts Medical School Worcester MA USA; ^2^ Department of Neurology University of Massachusetts Medical School Worcester MA USA; ^3^ Department of Microbiology and Physiological Systems University of Massachusetts Medical School Worcester MA USA

**Keywords:** adeno‐associated virus, glioblastoma, interferon‐beta, intracranial, syngeneic, xenograft

## Abstract

The highly invasive property of glioblastoma (GBM) cells and genetic heterogeneity are largely responsible for tumor recurrence after the current standard‐of‐care treatment and thus a direct cause of death. Previously, we have shown that intracranial interferon‐beta (IFN‐β) gene therapy by locally administered adeno‐associated viral vectors (AAV) successfully treats noninvasive orthotopic glioblastoma models. Here, we extend these findings by testing this approach in invasive human GBM xenograft and syngeneic mouse models. First, we show that a single intracranial injection of AAV encoding human IFN‐β eliminates invasive human GBM8 tumors and promotes long‐term survival. Next, we screened five AAV‐IFN‐β vectors with different promoters to drive safe expression of mouse IFN‐β in the brain in the context of syngeneic GL261 tumors. Two AAV‐IFN‐β vectors were excluded due to safety concerns, but therapeutic studies with the other three vectors showed extensive tumor cell death, activation of microglia surrounding the tumors, and a 56% increase in median survival of the animals treated with AAV/P2‐Int‐mIFN‐β vector. We also assessed the therapeutic effect of combining AAV‐IFN‐β therapy with temozolomide (TMZ). As TMZ affects DNA replication, an event that is crucial for second‐strand DNA synthesis of single‐stranded AAV vectors before active transcription, we tested two TMZ treatment regimens. Treatment with TMZ prior to AAV‐IFN‐β abrogated any benefit from the latter, while the reverse order of treatment doubled the median survival compared to controls. These studies demonstrate the therapeutic potential of intracranial AAV‐IFN‐β therapy in a highly migratory GBM model as well as in a syngeneic mouse model and that combination with TMZ is likely to enhance its antitumor potency.

**Abbreviations**
AAV: adeno‐associated viral vector
DAPI: 4′,6‐diamidino‐2‐phenylindole
DMSO: dimethyl sulfoxide
EGFP: enhanced green fluorescence protein
Fluc: firefly luciferase
GBM: glioblastoma
hIFN‐β: human interferon‐beta
JAK/STAT: Janus kinase/signal transducers and activators of transcription
mIFN‐β: mouse interferon‐beta
TMZ: temozolomide
WHO: World Health Organization

## 1 Introduction

Glioblastoma is the most common primary brain tumor (Preusser *et al*., 2011) as well as the most aggressive form (WHO grade IV) of glial cell tumors (Louis *et al*., 2007). The current standard‐of‐care treatment involves surgical resection, followed by radio‐ and chemotherapy with temozolomide (TMZ) (Hottinger *et al*., 2014). The tumor is characterized by highly invasive growth, neovascularization, and high mortality due to uncontrollable tumor recurrence. One of the primary causes for recurrence is the inability to surgically remove all tumor cells due to their ability to infiltrate normal tissue and migrate long distances. Moreover, GBM tumors develop exceptional resistance to radiation and temozolomide. As a result of these insidious properties of GBM, the median survival of patients with the current standard‐of‐care treatment is only 15–17 months from the time of diagnosis (Preusser *et al*., 2015; Stupp *et al*., 2009). Therefore, development of new therapies for GBM remains a high priority.

Interferon‐beta (IFN‐β) is a cytokine naturally secreted from cells upon viral infection, and it activates in target cells ~ 300 genes through the JAK/STAT signaling pathway (Schneider *et al*., 2014). In addition to immunomodulation during viral infections, IFN‐β is also reported to have antitumor properties that include direct cytostatic (Kaynor *et al*., 2002) and proapoptotic (Juang *et al*., 2004) effects, as well as indirect effects such as anti‐angiogenesis (Streck *et al*., 2005; Xiao *et al*., 2012), immune stimulation (Wolpert *et al*., 2015; Yang *et al*., 2014), and drug sensitization (Kase *et al*., 1993). A phase I study showed that using repeated intravenous delivery of recombinant IFN‐β as an adjuvant to the standard‐of‐care treatment could moderately improve the survival in patients without any severe side effects (Motomura *et al*., 2011). However, the effectiveness of this approach is likely limited by the short half‐life of IFN‐β in blood (Buchwalder *et al*., 2000).

Gene therapy can potentially overcome this limitation by genetically engineering cells in the target tissue to produce IFN‐β locally. Moreover, as IFN‐β is a secreted protein, the therapeutic effect is expected to extend beyond the transduced cells. Based on this concept, previously our group has shown that local intracranial delivery of a recombinant adeno‐associated virus (AAV) vector driving expression of human IFN‐β from a neuron‐specific promoter can eliminate orthotopic U87 human glioblastoma in athymic nude mouse model (Maguire *et al*., 2008). This study demonstrated the principle that AAV‐mediated local IFN‐β gene therapy could be useful for glioblastoma treatment. However, U87 tumor is noninvasive in nature unlike glioblastoma in patients where infiltration and migration are important features that contribute significantly to therapeutic failure. Therefore, in the current study we first sought to test the intracranial AAVrh8‐IFN‐β‐based therapeutic approach in a highly invasive human glioblastoma model (GBM8) (Wakimoto *et al*., 2009) implanted in athymic nude mice.

A limitation of therapeutic studies with human IFN‐β in xenograft GBM models is that they have to be conducted in immuno‐deficient athymic nude mice where human IFN‐β does not interact with mouse IFN‐β receptors (Harari *et al*., 2014). The complex interaction of IFN‐β with the immune system and tumor cells will determine the therapeutic outcome of an AAV‐IFN‐β strategy. Therefore, in a second set of experiments we tested the therapeutic efficacy of an AAV‐IFN‐β encoding the mouse cytokine in an orthotopic syngeneic mouse glioblastoma (GL261) model in normal C57BL/6J mice. Finally, we compared the therapeutic efficacy of AAV‐IFN‐β combined with the standard‐of‐care chemotherapeutic drug TMZ treatment in relation to chemotherapy alone.

## 2 Materials and methods

### 2.1 Cell culture, WST‐1 assay, and LDH assay

Human GBM8 glioblastoma cells (Wakimoto *et al*., 2009) were a kind gift from Dr. Samuel Rabkin (Massachusetts General Hospital, Boston, MA, USA). GBM8‐Fluc cells that constitutively express firefly luciferase were generated and grown as previously described (GuhaSarkar *et al*., 2016).

GL261 cells were purchased from the National Cancer Institute‐Frederick, Bethesda, MD, USA. The cells were grown in Dulbecco's modified Eagle's medium (Gibco, Grand Island, NY, USA) supplemented with 10% fetal bovine serum (Sigma‐Aldrich, St Louis, MO, USA), 1% penicillin–streptomycin (Gibco), and 1% L‐glutamine (Mediatech, Manassas, VA, USA).

WST‐1 assays (Roche, Westborough, MA, USA) were used to evaluate cell proliferation in the presence of recombinant human IFN‐β (hIFN‐β) protein or infection with AAV9/CB‐mIFN‐β for GBM8‐Fluc or GL261 cells, respectively. Adherent GBM8‐Fluc cells were cultured in plates precoated with laminin by overnight incubation with 10 μg·mL^−1^ laminin (Invitrogen, Carlsbad, CA, USA) at 37 °C. Studies were conducted in 12‐well dishes seeded with 200 000 cells per well 1 day before initiating the studies. The following day, the growth medium was replaced with fresh growth medium premixed with 100 IU of recombinant hIFN‐β protein (PeproTech, Rocky Hill, NJ, USA) supplemented every 72 h for GBM8 cells, or AAV9/CB‐mIFN‐β vector (at a dose of 100 000 vg per cell) for GL261 cells.

LDH assays (Roche) were used to assess cell death or cytotoxicity after recombinant hIFN‐β protein treatment to GBM8‐Fluc cells. Culturing of GBM8‐Fluc cells, plating, and IFN‐β treatments for this assay were similar to WST‐1 study. This time we tested the effect of treatment both with short‐term (every 3 h for 12 h) and long‐term (every 3 days for 9 days) exposure to hIFN‐β. To make sure that the assay is working, we treated the cells with 1% Triton X‐100 (BDH Chemicals, Westchester, PA, USA) as a positive control for the short‐term study. This cytotoxicity assay was performed in both the presence and absence of the growth factors in the media for the short‐term study and with growth factors for the long‐term study.

### 2.2 Animals

Six‐ to eight‐week‐old male athymic nude mice were obtained from the National Cancer Institute for the xenograft study with GBM8 cells. For the syngeneic tumor study, 6‐ to 8‐week‐old male C57BL/6J mice were used (Jackson Laboratory, Bar Harbor, ME, USA). The humane endpoint for the animals was defined as the loss of > 15% of maximum body weight or appearance of any neurological symptoms (such as hunched posture). All animal studies were approved by the Institutional Animal Care and Use Committee at the University of Massachusetts Medical School.

### 2.3 Tumor grafting in mouse brain

On the day of surgery, GBM8‐Fluc cells were dissociated and prepared as a single‐cell suspension in sterile Dulbecco's phosphate‐buffered saline (PBS) (Gibco), and 50 000 cells in 1 μL were injected stereotaxically into the left striatum of athymic nude mice at 0.2 μL·min^−1^. GL261 tumors were generated by stereotaxic injection of 10 000 GL261 cells in 1 μL into the left striatum of C57BL/6 mice using the same injection parameters. The stereotaxic coordinates for tumor implantation from bregma were as follows (in mm): AP: +0.5; ML: 2.0 (left); and DV from brain surface: −2.5.

### 2.4 AAV vector design, production, and delivery

For the xenograft study, AAVrh8/CBA‐hIFN‐β and a corresponding AAVrh8 vector without a transgene (empty vector) were used, as previously described (Maguire *et al*., 2008). For the studies in the syngeneic mouse tumor model, the following vectors were used: AAV9/CB‐mIFN‐β, AAV9/P2‐Int‐mIFN‐β, AAV9/hSyn1‐mIFN‐β, AAV9/Ple32‐mIFN‐β, and AAV9/Ple88‐mIFN‐β. These vectors contained mouse IFN‐β cDNA under the CB, P2‐Int, human Syn1, Ple32, or Ple88 promoters, respectively, and were packaged as AAV9. Detail descriptions of the promoters are provided in Table S1. For the GFP reporter study, the vectors used were as follows: AAV9/P2‐Int‐EGFP and AAV9/Ple88‐EGFP encoding the enhanced green fluorescence (GFP) protein. AAV vectors were delivered at the same site in the left striatum where tumors were implanted previously using the same stereotaxic coordinates and same flow rate of injection mentioned for the tumor grafting. The volume of injections for all AAV vectors was 2 μL, and PBS was used as the dilution medium.

### 2.5 Temozolomide treatment

Temozolomide (Selleckchem, Houston, TX, USA) powder was reconstituted in dimethyl sulfoxide (DMSO) (Thermo Fisher Scientific, Fair Lawn, NJ, USA) at a concentration of 20 mg·mL^−1^ and stored frozen at −80 °C as single‐use aliquots. Mice received 2.5 mg daily for five consecutive days from day 4 to day 8 (regimen 1) or day 10 to day 14 (regimen 2) after GL261 tumor implantation.

### 2.6 Live bioluminescence imaging

Live bioluminescence imaging of tumor‐associated firefly luciferase (Fluc) activity was performed using a Xenogen IVIS‐100 imaging system (PerkinElmer, Waltham, MA, USA) 3 min after intraperitoneal injection of 4.5 mg D‐luciferin in 150 μL of sterile PBS. Images were acquired using living image software (PerkinElmer).

### 2.7 Tissue RNA preparation and quantification of mIFN‐β transcripts

Total RNA was isolated using TRIzol (Invitrogen) reagent and Direct‐zol RNA MiniPrep kit (Zymo Research Corporation, Irvine, CA, USA) from 3‐mm striatal punches from the injected side of the brain. RNA was treated with TURBO DNase (Ambion, Foster City, CA, USA) for 30 min at 37 °C before using it for reverse transcription reaction with High‐Capacity RNA‐to‐cDNA kit (Applied Biosystems, Foster City, CA, USA). RT‐qPCR was performed using TaqMan gene Expression Master Mix (Applied Biosystems) with the following primers (IDT, Coralville, IA, USA) and probe (TIB Molbiol, Adelphia, NJ, USA) for bovine growth hormone polyadenylation signal (BGHpA) present on AAV transgene.

Forward primer sequence: 5′‐CCTCGACTGTGCC TTCTAG‐3′;
Reverse primer sequence: 5′‐TGCGATGCAATTT CCTCAT‐3′;
BGHpA probe: 6FAM‐TGCCAGCCATCTGTTG TTTGCC‐BBQ.

Mouse HPRT1 gene expression was used as an internal control to normalize all values (Assay ID: Mm00446968_m1; Applied Biosystems).

### 2.8 Mouse IFN‐β protein extraction from brain and detection by western blot

Mouse brain punches (3 mm diameter) were taken from the injection sites 1 month post‐AAV9/mIFN‐β injections. Tissues were collected into microcentrifuge tubes and frozen immediately in a dry ice/2‐methyl butane bath and stored at −80 °C. Protein extracts for western blot were prepared from frozen tissue pieces by bead lysis using T‐PER tissue lysis buffer (Thermo Fisher Scientific) supplemented with c*O*mplete Mini Protease Inhibitor cocktail (Roche, Indianapolis, IN, USA). Tissue lysates were centrifuged at 12 000 ***g*** for 1 min at 4 °C and total protein in the supernatant quantified using the Bradford protein assay (Bio‐Rad, Hercules, CA, USA). Total protein (60 μg) was separated by polyacrylamide gel electrophoresis in 4–20% Mini‐PROTEAN TGX gels (Bio‐Rad) and transferred to nitrocellulose membrane. Primary antibodies used in western blots were as follows: rabbit polyclonal anti‐mouse IFN‐β antibody (AB2215; EMD Millipore, Billerica, MA, USA, dilution 1 : 250) and mouse monoclonal anti‐‐β‐actin antibody (Sigma‐Aldrich, dilution 1 : 1000). Secondary antibodies used were as follows: horse radish peroxidase (HRP)‐conjugated ECL donkey anti‐rabbit IgG (GE Healthcare, Westborough, MA, USA, dilution 1 : 10 000) and HRP‐conjugated ECL sheep anti‐mouse IgG (GE Healthcare, dilution 1 : 10 000). Pierce ECL Western Blotting Substrate (Thermo Fisher Scientific) was used for detection, and X‐ray films were exposed for 30 min and 30 s for mIFN‐β and β‐actin detection, respectively. Two independent protein preparations were used for each sample.

### 2.9 Histological analysis

**Figure 1:**
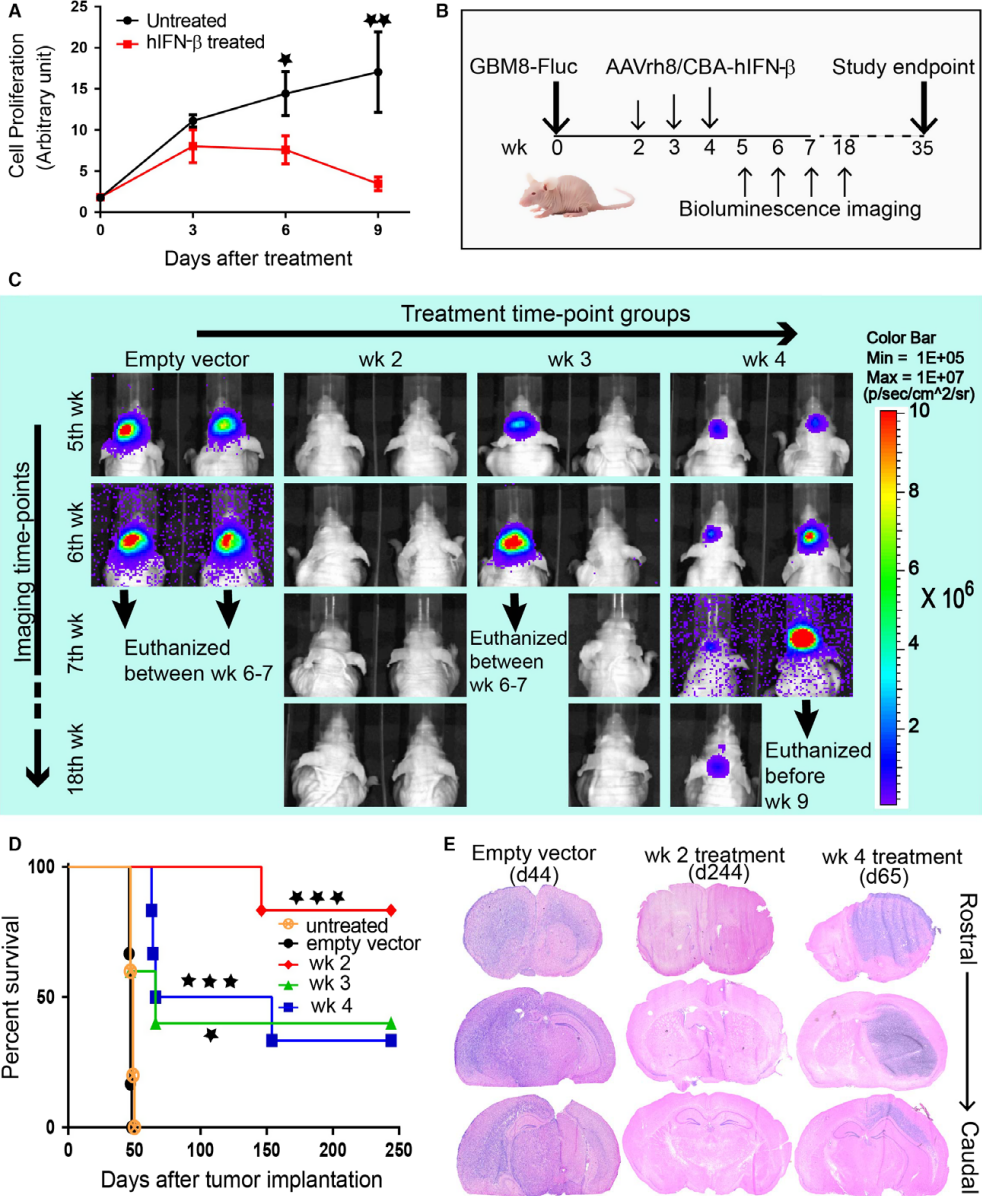
Local intracranial injection of AAVrh8/CBA‐hIFN‐β improves survival of GBM8‐Fluc‐implanted athymic nude mice. (A) WST‐1 assay shows the effect of recombinant hIFN‐β protein treatment on GBM8‐Fluc cell proliferation in culture. GBM8‐Fluc cells were treated with 100 IU recombinant hIFN‐β every 72 h. Unpaired Student's *t*‐test results: *P < 0.05; **P < 0.01. (B) Schematic diagram showing timing of tumor implantation, AAVrh8/CBA‐hIFN‐β treatment, and bioluminescence imaging. (C) Representative images of tumor‐associated bioluminescence signal in live mice from different groups. Two randomly chosen animals from each treatment group are shown. (D) Kaplan–Meier survival curves for treatment at different time‐points after tumor implantation: week 2 (red curve), week 3 (green curve), and week 4 (blue curve). Empty vector control (black curve) was injected at week 2. Another control group was left untreated (orange curve). Number of animals per group (*n*) = 5 for untreated and week 3 treatment groups. For all other groups, *n* = 6. Log‐rank test (compared to empty vector group): *P < 0.05; ***P < 0.001. (E) Representative rostral–caudal brain sections at the experimental endpoint for week 2 treatment (day 244), and humane endpoints for empty vector (day 44) and week 4 treatment (day 65). Abbreviations: wk, weeks post‐tumor implantation; d, days post‐tumor implantation.

**Figure 2:**
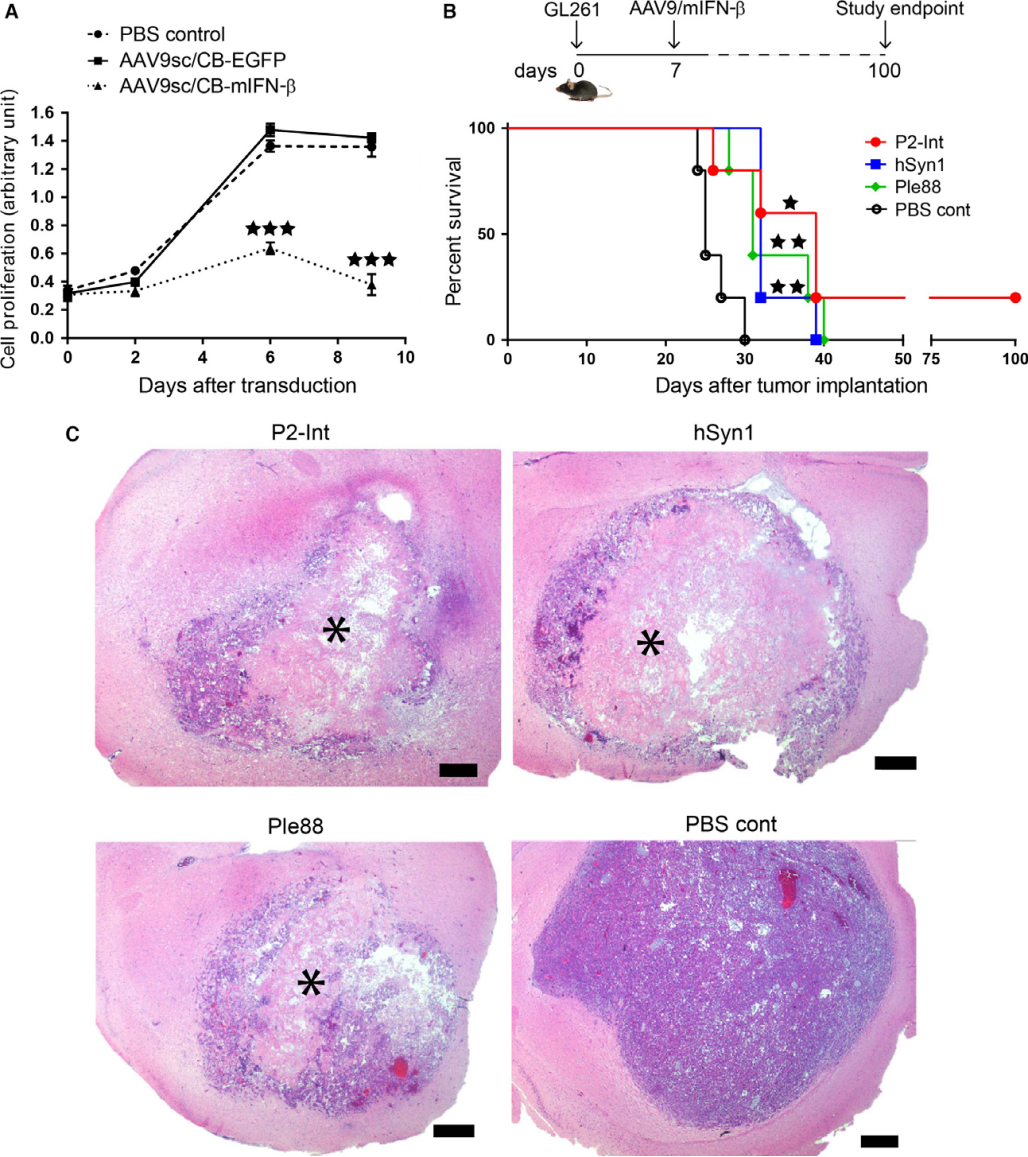
Local intracranial injection of AAV9‐mIFN‐β improves survival of GL261 tumor‐bearing mice. (A) WST‐1 assay shows the effect of AAV9/CB‐mIFN‐β treatment (MOI = 10^5^) on GL261 cell proliferation in culture. Controls were treated with AAV9/CB‐EGFP vector or PBS. Unpaired Student's *t*‐test results: ***P < 0.001. (B) Experimental design (top) and Kaplan–Meier survival curves (bottom) for therapeutic efficacy study in GL261‐implanted C57BL/6J mice treated with three different AAV9 vectors expressing mIFN‐β under different promoters: P2‐Int (red curve), hSyn1 (blue curve), and Ple88 (green curve). PBS was injected as control (black curve). Number of animals per group (*n*) = 5. Log‐rank test results: *P < 0.05; **P < 0.01. (C) Representative pictures of tumors at the humane endpoints from different vector treatment groups and PBS control. Asterisks indicate the site of extensive cell death after treatment. Scale bar represents 500 μm.

**Figure 3:**
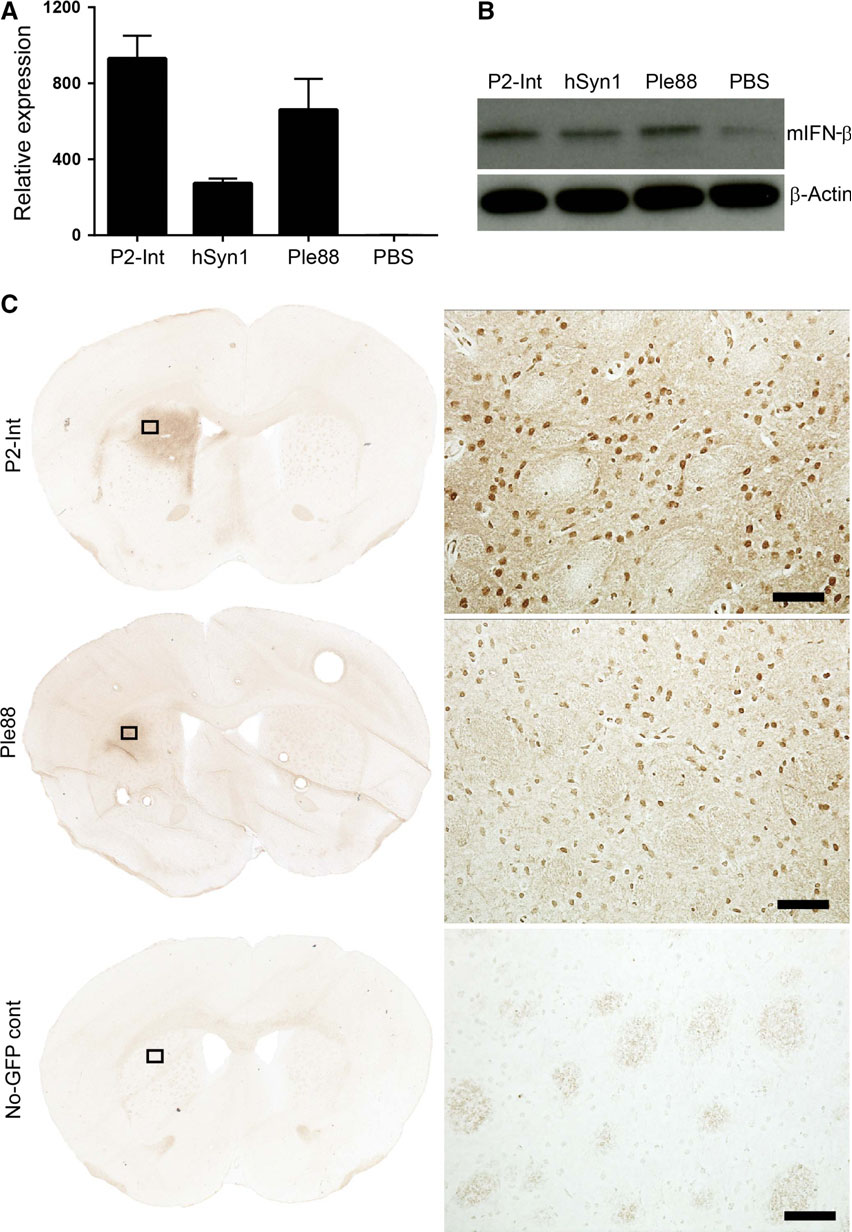
Transgene expression in brain after AAV9‐mediated intracranial delivery in mouse striatum. (A) RT‐qPCR analysis of AAV vector‐derived transgene mRNA. Punches were taken 1 month after intrastriatal injection of 3 × 10^10^ gc of AAV9 vectors encoding mIFN‐β gene under P2‐Int, hSyn1, or Ple88 promoters. (B) Western blot analysis of mIFN‐β expression from striatal punches of injected sites as described in A. (C) Distribution of EGFP‐positive cells in the striatum 1 month after injection of AAV9‐P2‐Int‐EGFP or AAV9‐Ple88‐EGFP vectors. An AAV9/P2‐int‐mIFN‐β‐injected brain was used as a no‐EGFP control. Corresponding magnified views of the areas marked with black boxes in the left panel are shown on the right. Scale bars represent 50 μm.

**Figure 4:**
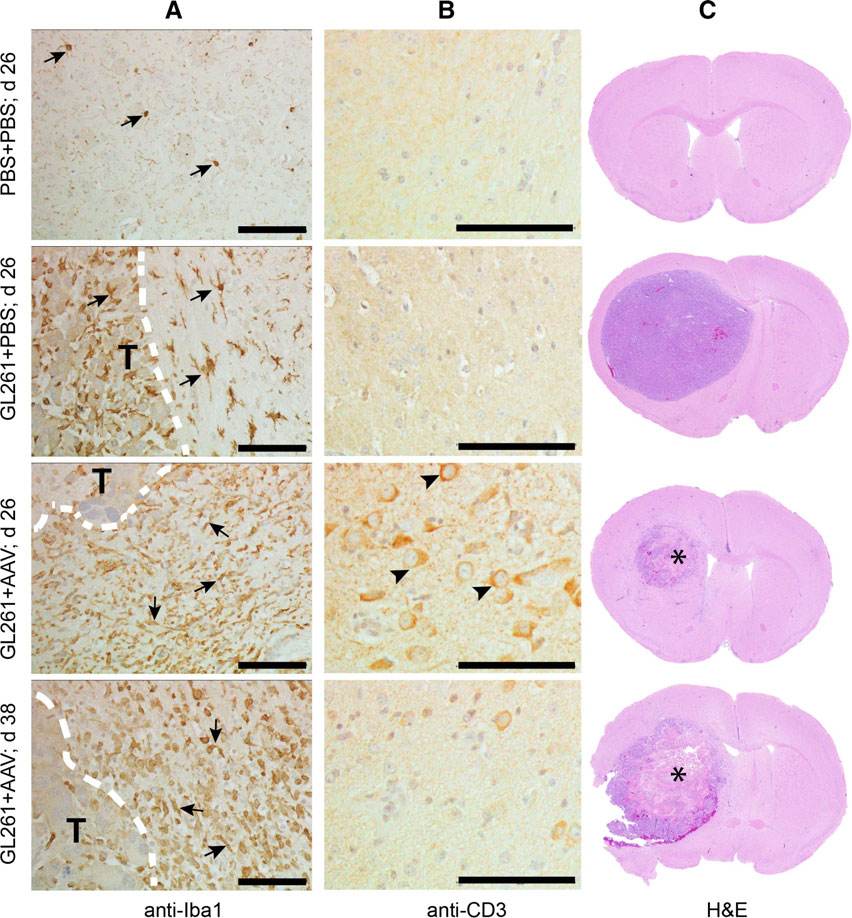
Histological analysis of mouse brains treated with AAV9/P2‐Int‐mIFN‐β. Representative brain sections immune‐stained with antibodies to (A) Iba1 or (B) CD3 to detect the presence of tumor‐associated microglia/macrophages and T cells, respectively. White discontinuous lines in (A) indicate the border between tumor and normal brain. The letter ‘T’ indicates the tumor side of the border. Arrows point to the Iba1+ cells. Arrowheads in (B) point to the CD3+ cells. (C) Corresponding hematoxylin–eosin‐stained sections. Asterisks indicate the site of extensive cell death after treatment. (A–C) Top row shows brains that received PBS in place of tumor cells and treatment. Bottom three rows show animal brains that received PBS or AAV9/P2‐Int‐mIFN‐β 7 days after tumor implantation, and collected at day 26, or at the respective humane endpoint. Scale bars represent 100 μm. Abbreviation: d, days post‐tumor implantation.

**Figure 5:**
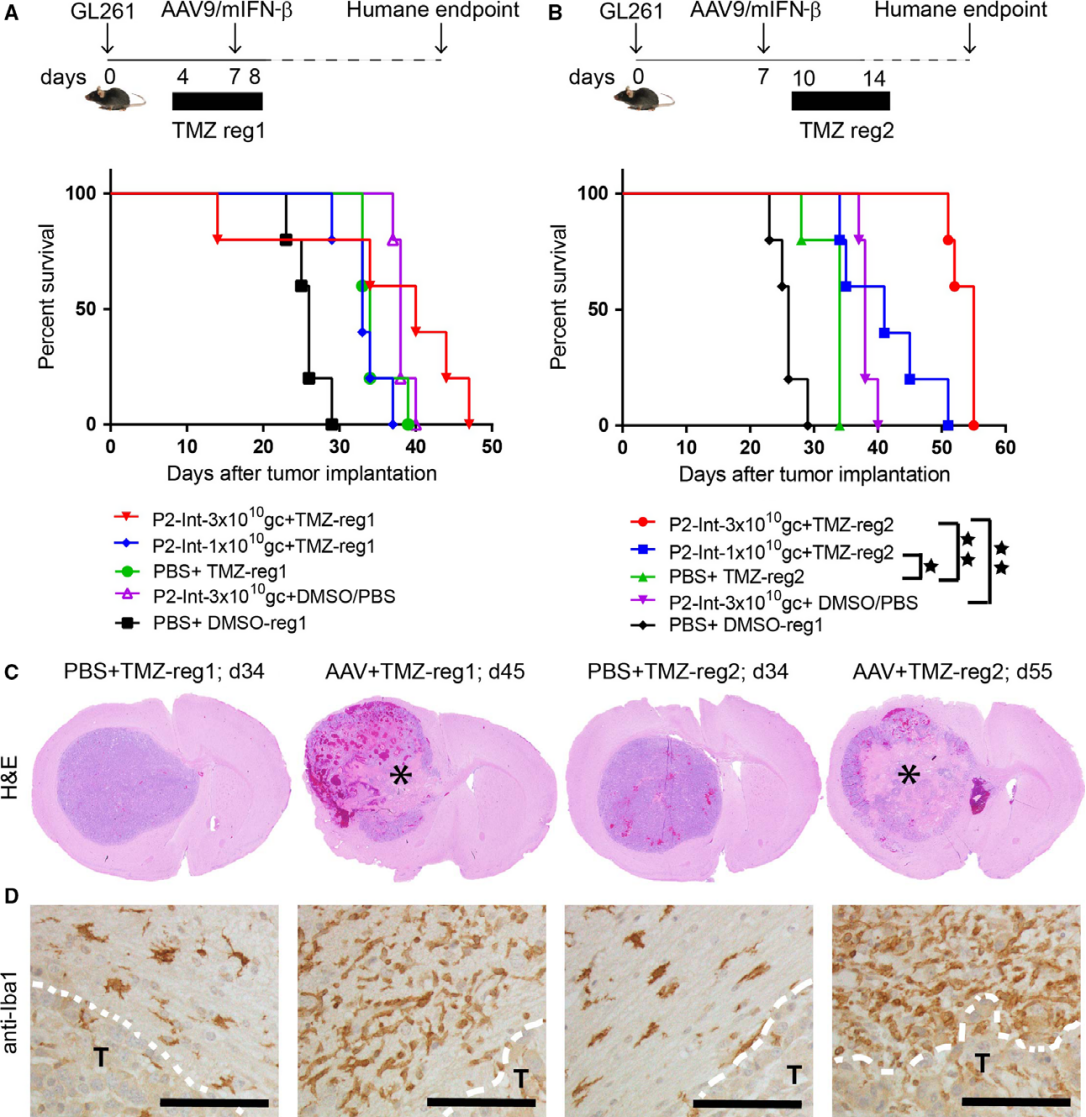
Combination of AAV9/P2‐Int‐mIFN‐β with temozolomide improves therapeutic efficacy. (A‐B) Schematic diagram of experimental paradigms (top) and Kaplan–Meier survival curves (bottom) for the therapeutic efficacy study of AAV9/P2‐Int‐mIFN‐β and temozolomide (TMZ) combined treatment. Two TMZ regimens were tested: daily 2.5 mg TMZ administered from day 4 to day 8 in regimen 1 (reg1) or day 10 to day 14 in regimen 2 (reg2). Diluted DMSO (1 : 3 in PBS) was used as control for TMZ and PBS was used as control for AAV treatment. The survival curve for the (PBS + diluted DMSO) control group (black line) was used for comparison both in A and in B. Log‐rank test results: *P < 0.05; **P < 0.01. Animals per group (*n*) = 5. (C) Hematoxylin–eosin staining of GL261 tumor‐bearing mouse brains at the humane endpoints. Asterisks indicate the site of extensive cell death after treatment. (D) Images show brain sections stained for the microglia marker Iba1. White discontinuous line indicates the border between tumor and normal tissue. The letter ‘T’ indicates the tumor location. Scale bars represent 100 μm. Abbreviations: reg, TMZ dose regimen; d, days post‐tumor implantation.

For histological analysis, we used 20‐μm cryosections (Fig. 1) or 5‐μm paraffin sections (Figs 2, 3, 4, 5) of mouse brains. Mayer's hematoxylin (Sigma‐Aldrich) and eosin Y alcoholic solution with phloxine (Sigma‐Aldrich) were used for histological staining. The primary antibodies used for immunohistochemistry were rabbit monoclonal anti‐GFP (G10362; Invitrogen, dilution 1 : 1000), rabbit polyclonal anti‐Iba1 (019‐19741; Wako, Cape Charles, VA, USA, dilution 1 : 500), and rabbit polyclonal anti‐CD3 (Abcam, Cambridge, MA, USA, dilution 1 : 100). Biotinylated goat anti‐rabbit IgG (Vector Labs, Burlingame, CA, USA, dilution 1 : 1000) was used as secondary antibody. VECTASTAIN Elite ABC Kit (Vector Labs) and DAB substrate kit (Vector Labs) were used for immunohistochemical detection. For immunofluorescence staining, the primary antibodies used were rabbit polyclonal anti‐GFP (A11122; Invitrogen, dilution 1 : 1000) and mouse monoclonal anti‐NeuN (MAB377; EMD Millipore, 1 : 500). The secondary antibodies used were Alexa Fluor 488‐conjugated F(ab′)2 goat anti‐rabbit IgG (A‐11070; Thermo Fisher Scientific, dilution 1 : 1000) and Alexa Fluor 594 goat anti‐mouse (A‐11020; Thermo Fisher Scientific, dilution 1 : 2000). Sections were counterstained with nuclear stain 4′,6‐diamidino‐2‐phenylindole (DAPI) (Thermo Fisher Scientific, 0.1 μg·mL^−1^). Three or four different brains and three individual sections from each brain were analyzed for all histological data, and the images shown are representative. Leica DM5500 B microscope (Leica Microsystems, Buffalo Grove, IL, USA) was used for all image acquisitions, and Adobe Photoshop CS6 (Adobe Systems, San Jose, CA, USA) was used for image processing.

### 2.10 Graphs and statistical analysis

Graphs and statistical analyses were performed in prism 6 (GraphPad Software, La Jolla, CA, USA). Kaplan–Meier survival curves were analyzed using Mantel–Cox log‐rank tests. Unpaired two‐tailed *t*‐test was performed for statistical analysis of the bar graphs. P values were indicated as *P < 0.05, **P < 0.01, and ***P < 0.001.

## 3 Results

### 3.1 Single intracranial injection of AAV‐hIFN‐β successfully treats tumors in invasive glioblastoma xenograft model

Treatment of GBM8‐Fluc cells in culture with recombinant human IFN‐β over a 9‐day period resulted in significant inhibition of proliferation by day 6 (Fig. 1A). However, we did not see any significant cell death for IFN‐β‐treated cells in this period (Fig. S1). Next, we tested the therapeutic efficacy of intracranial administration of 3 × 10^10^ gc AAVrh8/CBA‐hIFN‐β in GBM8 tumor‐bearing mice at 2, 3, or 4 weeks after tumor implantation (Fig. 1B). Tumor growth was assessed weekly by live imaging of tumor‐associated bioluminescence signal (TABS) starting at 5 weeks after tumor implantation, a time when there was no detectable TABS in week 2 treatment group animals, whereas all animals in the empty vector control group showed high signals. In week 3 and week 4 treatment groups, there was considerable variability in TABS among animals (Fig. 1C). An 8‐month study showed that 83.3% of mice in the week 2 group survived until the experimental endpoint, whereas all animals in the control groups (untreated and empty vector) were euthanized on or before day 50 after tumor implantation with median survivals of 47–49 days. Consistent with the variability in TABS, a smaller percentage of animals in week 3 (33.3%) and week 4 (40%) groups survived until the experimental endpoint with median survivals of 66 and 110 days, respectively (Fig. 1D). Histological analysis of brains at the endpoint revealed diffuse ipsilateral tumors and long‐distance migration to the contralateral hemisphere in animals from the control groups. In contrast, there was no evidence of tumors in animals that survived to the experimental endpoint at 8 months. Animals from week 3 and week 4 treatment groups that reached the humane endpoint showed tumors of varying sizes and spread. Of note is that in several animals large tumors were found in the contralateral hemisphere and these appeared less diffuse than tumors in the control groups (Fig. 1E).

### 3.2 Single intracranial injection of AAV‐mIFN‐β improves survival of normal mice with syngeneic GL261 brain tumors

Next, we tested the therapeutic efficacy of intracranial AAV‐IFN‐β gene therapy in the GL261 brain tumor model in C57BL/6J mice. For these studies, all AAV vectors encoding mouse IFN‐β (mIFN‐β) as the human cytokine do not have antiproliferative properties in mouse tumors (Harari *et al*., 2014). Transduction of GL261 cells in culture with AAV9‐mIFN‐β resulted in significant inhibition of proliferation by day 6 compared to both PBS and AAV9‐EGFP controls (Fig. 2A).

As IFN‐β is a potent cytokine, we tested five different AAV9 vectors encoding mIFN‐β under different promoters (Table S1) for safety and antitumor effect in a pretreatment experimental model. AAV9 vectors (5 × 10^9^ gc) were injected in the striatum 2 weeks prior to injection of GL261 tumor cells in the same stereotaxic coordinates and the effect on survival analyzed. Kaplan–Meier survival curves (Fig. S2) showed that groups treated with AAV9 vectors carrying Ple32 and CB promoters had lower median survival (21 days) than the PBS control group (25 days), suggestive of toxicity. Hence, these two vectors were excluded from further therapeutic studies. The other three groups treated with AAV9 vectors carrying promoters P2‐Int, hSyn1, and Ple88 showed small improvements in median survival to 31, 31, and 33 days, respectively (Fig. S2). These three vectors were selected for further studies.

Tumor‐bearing mice received intrastriatal injections of the AAV9‐mIFN‐β vectors selected above (3 × 10^10^ gc), or PBS, at the site of GL261 tumor cell implantation. Kaplan–Meier survival curves showed moderate but statistically significant improvements in survival for all AAV treatment groups. Median survivals were improved to 31, 32, and 39 days for the Ple88, hSyn1, and P2‐Int groups, respectively, from 25 days in PBS control (Fig. 2B). Histological analysis revealed extensive cell death in the tumor center in AAV treatment groups unlike the more uniform appearance of tumors in the PBS control group (Fig. 2C).

### 3.3 Transgenes are expressed in brain after AAV9 gene delivery to mouse striatum

The therapeutic effect of AAV9‐mIFN‐β vectors was likely mediated by expression of IFN‐β as its mRNA (Fig. 3A) and protein (Fig. 3B) levels were elevated in the injected striatum compared to PBS controls. The hSyn1 promoter is known to drive neuron‐specific transgene expression in mouse brain after AAV‐mediated delivery (Lukashchuk *et al*., 2016). Striatal injection of AAV9/P2‐Int‐EGFP and AAV9/Ple88‐EGFP vectors (3 × 10^10^ gc) resulted in efficient distribution and transduction of cells of primarily neuronal phenotype (Figs 3C and S3).

### 3.4 Histological analysis of microglia/macrophage activation and T‐cell infiltration in tumor‐bearing brain after AAV9/P2‐Int‐mIFN‐β treatment

As tumor‐associated microglia/macrophages (TAMs) and T cells are important components of the immune system shown to influence brain tumor growth (Kmiecik *et al*., 2013; Li and Graeber, 2012; See *et al*., 2015), we analyzed the presence of these cells in tumor‐bearing brains after treatment with AAV9/P2‐Int‐mIFN‐β vector, as the corresponding treatment cohort had the longest median survival (Fig. 2B). Activated TAMs (amoeboid Iba1+ cells) were readily apparent in all tumor‐implanted brains, both AAV‐treated and PBS‐treated, compared to brains without tumor, where we saw only resting Iba1+ cells (fine ramified structure). However, in AAV9/P2‐Int‐mIFN‐β‐treated brains there were noticeably higher numbers of activated TAMs in the brain around the tumor border compared to the PBS‐treated control (Fig. 4A). CD3+ T cells were present in AAV‐treated brains in normal brain parenchyma near the tumor border at an early time‐point (day 26 after tumor implantation) in contrast to PBS‐treated controls where there was no evidence of CD3+ cells. However, CD3+ T cells were no longer found at the humane endpoint (day 38) for the AAV‐treated group (Fig. 4B). Interestingly, we observed that at the time of T‐cell infiltration (day 26), the tumor size in AAV‐treated brains was markedly smaller when compared to PBS‐treated control and the treated tumors already showed considerable amount of cell death at the center (Fig. 4C).

### 3.5 AAV9/P2‐Int‐mIFN‐β and temozolomide combination therapy improves survival over each treatment modality alone

Temozolomide (TMZ) is the standard‐of‐care chemotherapeutic drug used in conjunction with radiation following neurosurgical resection. Here, we sought to determine whether the combination of AAV9/P2‐Int‐mIFN‐β with TMZ has increased potency compared to each therapy alone. However, as TMZ compromises DNA replication, which is necessary for conversion of AAV to a transcriptionally active double‐stranded genome, it is possible that the combination would compromise the therapeutic effect of IFN‐β gene therapy. To assess this possibility, two regimens were tested where GL261‐implanted animals were treated with TMZ for five consecutive days starting 3 days before (regimen 1) or 3 days after (regimen 2) AAV treatment. Two doses (1 × 10^10^ or 3 × 10^10^ gc) of AAV9/P2‐int‐mIFN‐β were tested. Regimen 1 provided no survival benefit compared to either modality alone (Fig. 5A), while the combination of TMZ with AAV in regimen 2 provided a significant improvement in survival (Fig. 5B). In regimen 2, the median survival for TMZ or AAV9/P2‐Int‐mIFN‐β (at 3 × 10^10^ gc) alone was 34 and 38 days, respectively, while the median survival for the combination therapy was 41 and 55 days for AAV doses of 1 × 10^10^ and 3 × 10^10^ gc, respectively. Overall, the median survival for combination therapy with TMZ regimen 2 and AAV9/P2‐Int‐mIFN‐β at 3 × 10^10^ gc more than doubled the median survival of the PBS + DMSO control group, where it was only 26 days.

Histopathological analysis of the brains in the TMZ/AAV combination groups showed extensive cell death at the tumor center compared to TMZ‐only‐treated groups (Fig. 5C). In addition, there were considerably more activated TAMs (amoeboid Iba1+ cells) in the brain around the tumor in the combination treatment groups compared to the ones treated with TMZ alone (Fig. 5D).

## 4 Discussion

Studies in the past have demonstrated the antitumor potential of IFN‐β protein on glioblastoma ranging from cell culture studies (Happold *et al*., 2014) to clinical trials (Motomura *et al*., 2011). But the short half‐life (Buchwalder *et al*., 2000) of recombinant IFN‐β is a major limiting factor in achieving greater therapeutic benefit. Gene therapy is an attractive approach to overcome this limitation. Several studies from our group (Maguire *et al*., 2008; Meijer *et al*., 2009) and others (Denbo *et al*., 2011; Streck *et al*., 2006) have demonstrated the potential of IFN‐β gene therapy for glioblastoma. But none of these studies has tested this approach in an orthotopic glioblastoma model that is truly invasive in nature. The current study, to the best of our knowledge, is the first that shows that local IFN‐β gene therapy can effectively treat highly invasive human glioblastoma in an orthotopic mouse xenograft model. This is also the first study where long‐term survival benefit was achieved in a highly invasive glioblastoma model using only a single local intracranial injection of AAV vectors. An interesting finding was that in week 3 and week 4, treated animals that succumbed to disease progression had tumors with distinct borders unlike the diffuse pattern observed for these invasive GBM8 tumors in PBS‐treated control animals. This suggests that IFN‐β may have antimigratory properties, but further studies are necessary to further investigate this unexpected finding.

In the current study, we also assessed the therapeutic potential of AAV‐IFN‐β gene therapy in a syngeneic mouse glioblastoma model. As IFN‐β interacts with its receptor in a species‐specific manner for its antiproliferation effect (Harari *et al*., 2014), we used mouse IFN‐β for treatment of this mouse tumor. While AAVrh8 was used for the xenograft study to be consistent with our previous study with xenograft orthotopic study (Maguire *et al*., 2008), we used AAV9 vectors in the syngeneic study as AAV9 is more clinically relevant than AAVrh8 as there are several ongoing clinical trials for neurological diseases using AAV9 (ClinicalTrials.gov identifiers: NCT02122952, NCT02725580, NCT02240407). We tested different promoters that were expected to be active in different cell types in the brain to design an AAV9‐mIFN‐β vector for optimal expression of IFN‐β. This was critical to reduce the risk of possible detrimental side effects related to continuous expression of IFN‐β in the brain, as became apparent for a subset of AAV vectors where survival of treated animals was shorter than that of PBS‐treated controls (Fig. S2). Nonetheless, the screening process identified several AAV9 vector designs that provided moderate survival benefit, and the histological appearance of tumors in AAV treatment groups with large centers devoid of cells indicates that mIFN‐β expression had a potent biological effect on the tumor. In these studies, mice reached the humane endpoint because of rapid body weight loss and appearance of neurological symptoms. In control animals, this was likely caused by the large tumor masses that occupy a substantial fraction of the ipsilateral hemisphere. However, the reason behind AAV‐treated animals reaching humane endpoint may have been caused by the residual tumor mass or by an inflammatory response triggered either by a direct effect of mIFN‐β on microglia or by the tumor cell death. Apparently exposure of microglia to IFN‐β triggers the secretion of neuroinflammatory mediators (TNF‐α, IL‐1‐β, IL‐6, or nitric oxide) (Kawanokuchi *et al*., 2004). This can potentially have detrimental effects on the nervous system in a mouse, which in turn could result in body weight loss in the long term and thus forcing humane euthanasia.

The notion that an inflammatory response may have been triggered by AAV‐mIFN‐β treatment is supported by the substantial increase in activated microglia (amoeboid Iba1+ cells) around tumors in treatment groups compared to controls. Nonetheless, gliomas are also known to secrete chemoattractant molecules (e.g., CSF1, SDF1, CX3CL1) that attract microglia/macrophages (Hambardzumyan *et al*., 2016). Presently, it is unknown how the increased numbers of tumor‐associated microglia/macrophages (TAMs) associated with AAV‐mIFN‐β treatment affected tumor growth.

Finally, we have also shown that AAV‐mIFN‐β gene therapy can be applied in tandem with the current standard care temozolomide treatment to significantly improve the survival benefit. Interestingly, we have observed that the temporal order of temozolomide and AAV treatment is an important factor in determining the added benefit from the combined therapy. We found that starting temozolomide prior to AAV abolished the potentiated therapeutic benefit observed when temozolomide was started 3 days after AAV injection (Fig. 5A,B). Although we did not study the mechanistic details, one possible reason for this observation could be that DNA alkylation caused by temozolomide interferes with the DNA synthesis necessary to generate transcriptionally active double‐stranded AAV genomes (McCarty, 2008). Presently, we are unable to exclude the possibility that TMZ may inhibit any number of the cellular pathways necessary for productive AAV gene expression. While further studies are needed to uncover the exact mechanism, the potential effect of TMZ on AAV gene transfer to CNS is an important finding for any future therapies involving AAV gene delivery and DNA‐alkylating agents. The detrimental effect of TMZ on AAV gene therapy may not be a significant limitation in clinical practice as chemotherapy usually starts 4 weeks after surgery in patients with glioblastoma (Mao *et al*., 2015; Stupp *et al*., 2005), and thus, local injection of AAV‐IFN‐β vectors in the brain tissue immediately after surgical resection of tumors would be a viable option.

The current study shows the intracranial AAV‐IFN‐β gene therapy approach to be effective in two different aggressive glioblastoma models. It has been successful to provide long‐term survival in the invasive human GBM xenograft model and more than doubled the median survival in the syngeneic GL261 mouse tumor model when used in combination with temozolomide therapy. Considering that the median survival of patients with glioblastoma is very short with the current standard‐of‐care treatment, the therapeutic benefit shown for intracranial AAV‐IFN‐β therapy would be a significant improvement. Moreover, the therapeutic impact of this gene therapy approach may be further refined by fine‐tuning IFN‐β expression using transcriptional and post‐transcriptional regulation to make transgene expression more specific for tumor cells or tumor microenvironment (Ruan *et al*., 2001; Wu *et al*., 2009; Yawata *et al*., 2011). Use of switchable promoters (Chtarto *et al*., 2003) could be an option to avoid possible toxicity from long‐term continuous expression of IFN‐β, and cotreatment of phosphodiesterase inhibitors could potentially minimize the possible neuroinflammatory side effects of IFN‐β treatment (Kawanokuchi *et al*., 2004).

## Author contributions

MS‐E conceived the study; DGS and MS‐E designed the experiments; DGS, JN, and QS executed the experiments; DGS and JN acquired data; DGS and MS‐E analyzed and interpreted the results and wrote the manuscript; and GG and MS‐E supervised the study, helped with important advices, and reviewed and edited the manuscript.

## Supporting information


**Fig. S1.** Lactate dehydrogenase cytotoxicity assay of GBM8‐Fluc cells after treatment with recombinant human IFN‐β protein.

**Fig. S2.** Pretreatment of GL261 tumor‐implanted C57BL/6J mice with AAV9 vectors encoding mIFN‐β genes driven under different promoters.

**Fig. S3.** Double immunofluorescence staining of brain tissue section for neuron marker and AAV‐transduced cells.

**Table S1.** Descriptions and sequences of promoters.
