# Supplementary figures and images for: Intracranial AAV‐IFN‐β gene therapy eliminates invasive xenograft glioblastoma and improves survival in orthotopic syngeneic murine model
Source: Mol Oncol. 2017 Jan 18;11(2):180–93. doi: 10.1002/1878-0261.12020 (PMC5288127; doi:10.1002/1878-0261.12020)

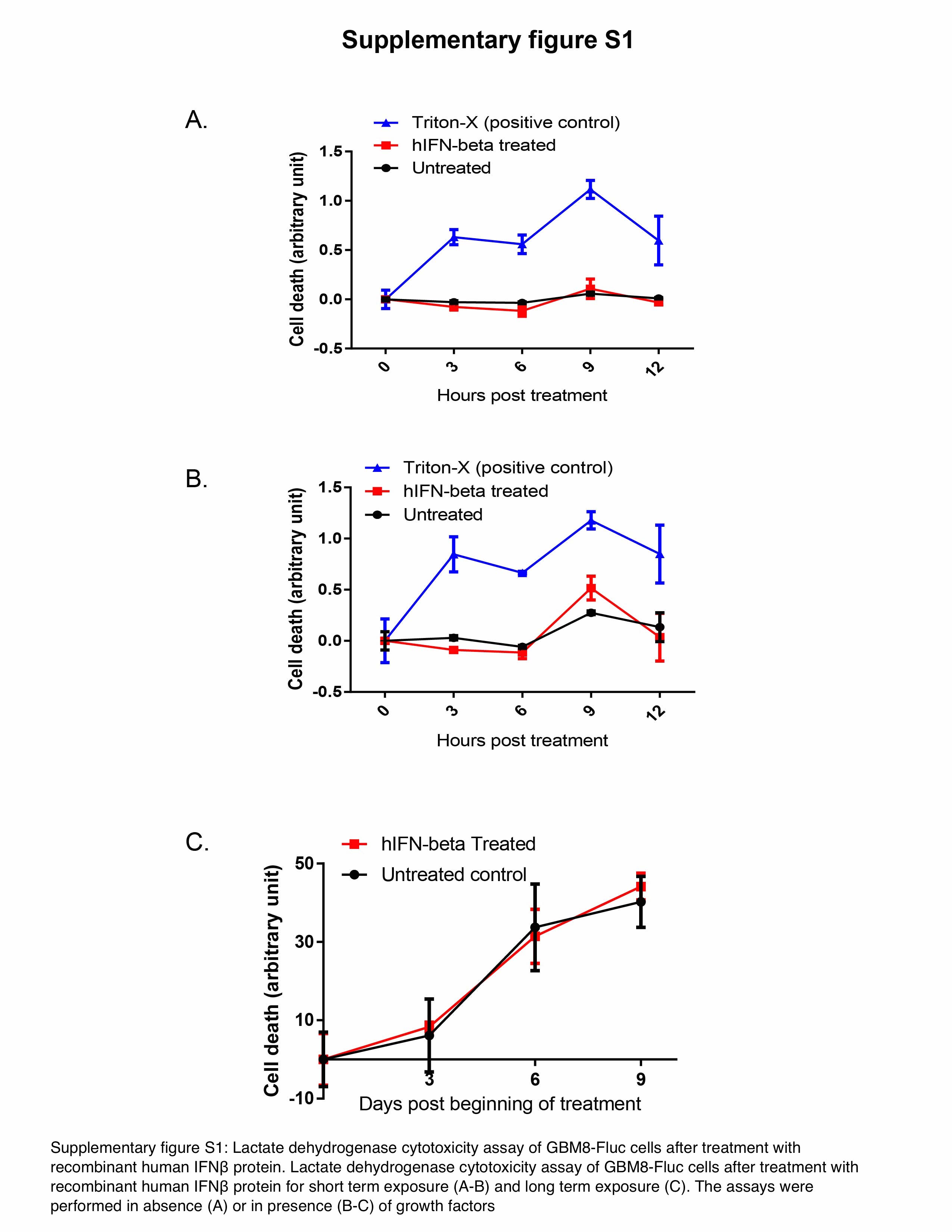

Supplement: Supplementary file 1 — Fig. S1. Lactate dehydrogenase cytotoxicity assay of GBM8‐Fluc cells after treatment with recombinant human IFN‐β protein. [file MOL2-11-180-s001.jpg]

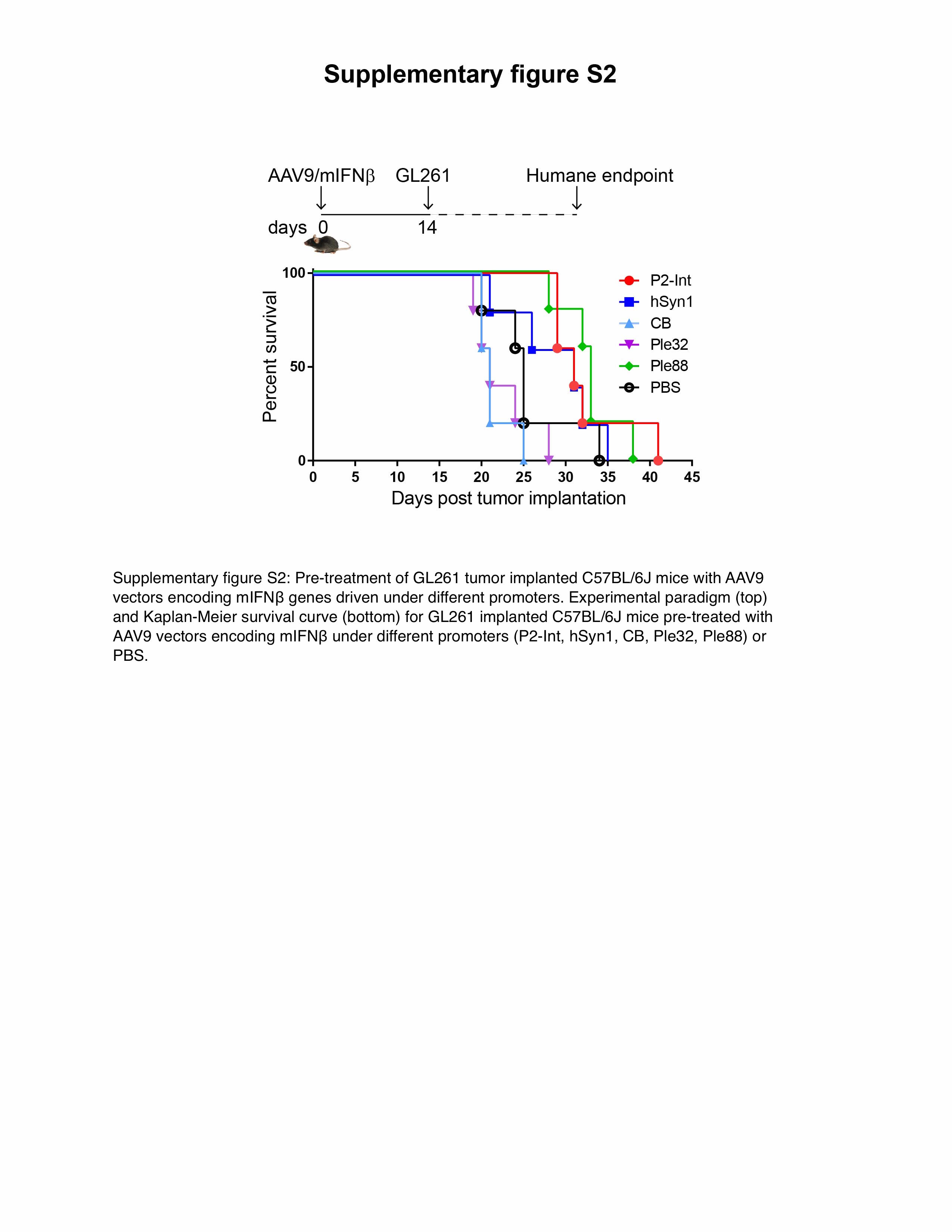

Supplement: Supplementary file 2 — Fig. S2. Pretreatment of GL261 tumor‐implanted C57BL/6J mice with AAV9 vectors encoding mIFN‐β genes driven under different promoters. [file MOL2-11-180-s002.jpg]

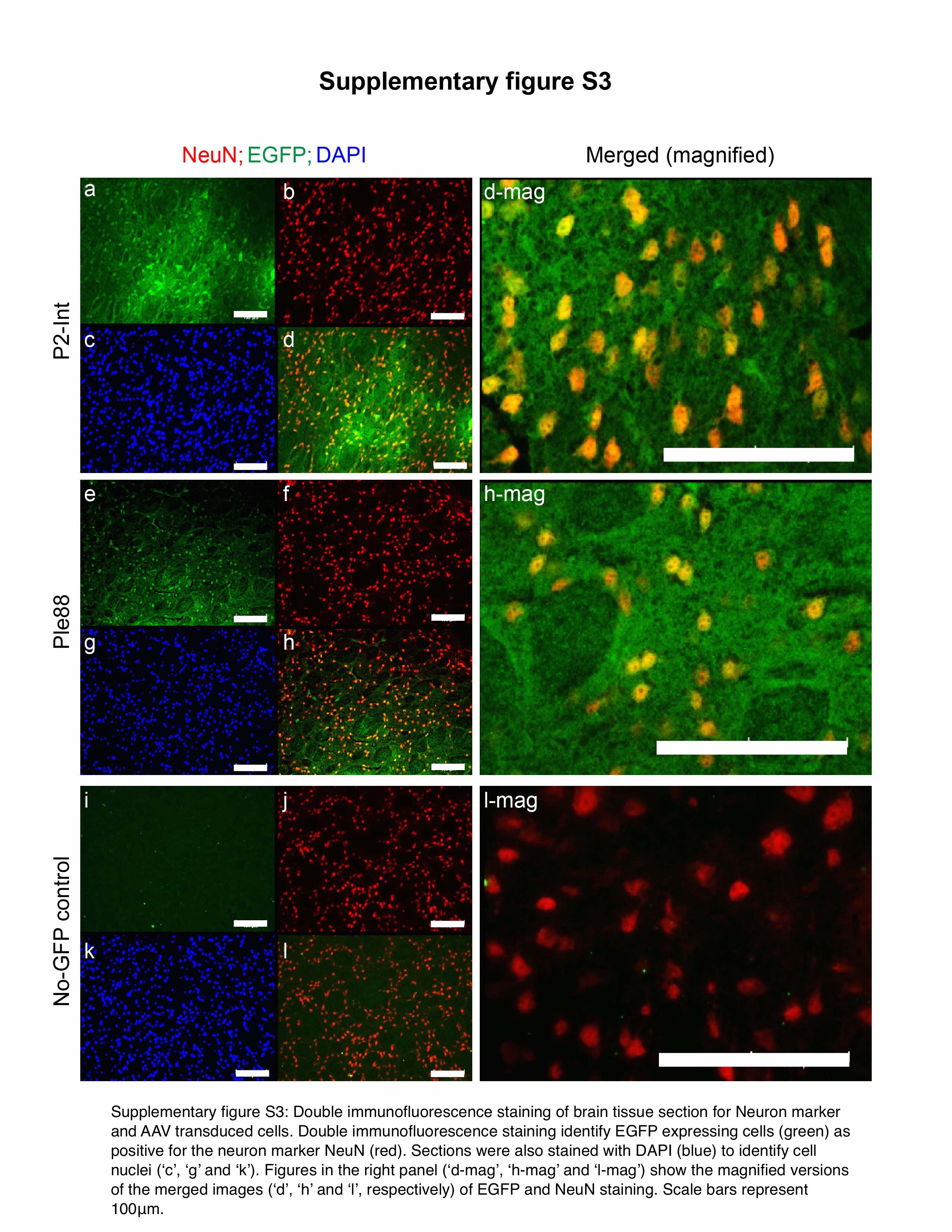

Supplement: Supplementary file 3 — Fig. S3. Double immunofluorescence staining of brain tissue section for neuron marker and AAV‐transduced cells. [file MOL2-11-180-s003.jpg]
