# Supplementary material for: Intracranial AAV‐IFN‐β gene therapy eliminates invasive xenograft glioblastoma and improves survival in orthotopic syngeneic murine model
Source: Mol Oncol. 2017 Jan 18;11(2):180–93. doi: 10.1002/1878-0261.12020 (PMC5288127; doi:10.1002/1878-0261.12020)
Supplement: Supplementary file 4 — Table S1. Descriptions and sequences of promoters. [file MOL2-11-180-s004.docx]

**Supplementary Table T1**

**Promoter descriptions:**

| **Promoter name** | **CBA** |
| --- | --- |
| **Description** | Chicken beta actin promoter, which is also known as CAG promoter. It involves cytomegalovirus immediate early (CMV IE) enhancer, chicken beta-actin first exon and a chimeric intron, which includes chicken beta-actin first intron and a splice acceptor site from the rabbit beta-globin gene. |
| **Sequence** | CGCGTCGACATTGATTATTGACTAGTTATTAATAGTAATCAATTACGGGGTCATTAGTTCATAGCCCATATATGGAGTTCCGCGTTACATAACTTACGGTAAATGGCCCGCCTGGCTGACCGCCCAACGACCCCCGCCCATTGACGTCAATAATGACGTATGTTCCCATAGTAACGCCAATAGGGACTTTCCATTGACGTCAATGGGTGGAGTATTTACGGTAAACTGCCCACTTGGCAGTACATCAAGTGTATCATATGCCAAGTACGCCCCCTATTGACGTCAATGACGGTAAATGGCCCGCCTGGCATTATGCCCAGTACATGACCTTATGGGACTTTCCTACTTGGCAGTACATCTACGTATTAGTCATCGCTATTACCATGGTCGAGGTGAGCCCCACGTTCTGCTTCACTCTCCCCATCTCCCCCCCCTCCCCACCCCCAATTTTGTATTTATTTATTTTTTAATTATTTTGTGCAGCGATGGGGGCGGGGGGGGGGGGGGGGCGCGCGCCAGGCGGGGCGGGGCGGGGCGAGGGGCGGGGCGGGGCGAGGCGGAGAGGTGCGGCGGCAGCCAATCAGAGCGGCGCGCTCCGAAAGTTTCCTTTTATGGCGAGGCGGCGGCGGCGGCGGCCCTATAAAAAGCGAAGCGCGCGGCGGGCGGGAGTCGCTGCGACGCTGCCTTCGCCCCGTGCCCCGCTCCGCCGCCGCCTCGCGCCGCCCGCCCCGGCTCTGACTGACCGCGTTACTCCCACAGGTGAGCGGGCGGGACGGCCCTTCTCCTCCGGGCTGTAATTAGCGCTTGGTTTAATGACGGCTTGTTTCTTTTCTGTGGCTGCGTGAAAGCCTTGAGGGGCTCCGGGAGCTAGAGCCTCTGCTAACCATGTTCATGCCTTCTTCTTTTTCCTACAGCTCCTGGGCAACGTGCTGGTTATTGTGCTGTCTCATCATTTTGGCAAAGAATTCCTCGAAGATCCGAAGGGGTTCA |

| **Promoter name** | **CB** |
| --- | --- |
| **Description** | Chicken beta actin with CMV IE enhancer, but no introns |
| **Sequence** | CGCGTCGACATTGATTATTGACTAGTTATTAATAGTAATCAATTACGGGGTCATTAGTTCATAGCCCATATATGGAGTTCCGCGTTACATAACTTACGGTAAATGGCCCGCCTGGCTGACCGCCCAACGACCCCCGCCCATTGACGTCAATAATGACGTATGTTCCCATAGTAACGCCAATAGGGACTTTCCATTGACGTCAATGGGTGGAGTATTTACGGTAAACTGCCCACTTGGCAGTACATCAAGTGTATCATATGCCAAGTACGCCCCCTATTGACGTCAATGACGGTAAATGGCCCGCCTGGCATTATGCCCAGTACATGACCTTATGGGACTTTCCTACTTGGCAGTACATCTACGTATTAGTCATCGCTATTACCCACGTTCTGCTTCACTCTCCCCATCTCCCCCCCCTCCCCACCCCCAATTTTGTATTTATTTATTTTTTAATTATTTTGTGCAGCGATGGGGGCGGGGGGGGGGGGCGCGCGCCAGGCGGGGCGGGGCGGGGCGAGGGGCGGGGCGGGGCGAGGCGGAGAGGTGCGGCGGCAGCCAATCAGAGCGGCGCGCTCCGAAAGTTTCCTTTTATGGCGAGGCGGCGGCGGCGGCGGCCCTATAAAAAGCGAAGCGCGCGGCGGG |

| **Promoter name** | **Ple88** |
| --- | --- |
| **Description** | Originally derived from human Glial fibrillary acidic protein promoter, sourced from Addgene plasmid #29176: pEMS1375, described in Portales-Casamar et al.^1^. |
| **Sequence** | GGCCGGCCCAGATGTGACTAGAGCCTAAGGAGCTCCCACCTCCCTCTCTGTGCTGGGACTCACAGAGGGAGACCTCAGGAGGCAGTCTGTCCATCACATGTCCAAATGCAGAGCATACCCTGGGCTGGGCGCAGTGGCGCACAACTGTAATTCCAGCACTTTGGGAGGCTGATGTGGAAGGATCACTTGAGCCCAGAAGTTCTAGACCAGCCTGGGCAACATGGCAAGACCCTATCTCTACAAAAAAAGTTAAAAAATCAGCCACGTGTGGTGACACACACCTGTAGTCCCAGCTATTCAGGAGGCTGAGGTGAGGGGATCACTTAAGGCTGGGAGGTTGAGGCTGCAGTGAGTCGTGGTTGCGCCACTGCACTCCAGCCTGGGCAACAGTGAGACCCTGTCTCAAAAGACAAAAAAAAAAAAAAAAGAACATATCCTGGTGTGGAGTAGGGGACGCTGCTCTGACAGAGGCTCGGGGGCCTGAGCTGGCTCTGTGAGCTGGGGAGGAGGCAGACAGCCAGGCCTTGTCTGCAAGCAGACCTGGCAGCATTGGGCTGGCCGCCCCCCAGGGCCTCCTCTTCATGCCCAGTGAATGACTCACCTTGGCACAGACACAATGTTCGGGGTGGGCACAGTGCCTGCTTCCCGCCGCACCCCAGCCCCCCTCAAATGCCTTCCGAGAAGCCCATTGAGCAGGGGGCTTGCATTGCACCCCAGCCTGACAGCCTGGCATCTTGGGATAAAAGCAGCACAGCCCCCTAGGGGCTGCCCTTGCTGTGTGGCGCCACCGGCGGTGGAGAACAAGGCTCTATTCAGCCTGTGCCCAGGAAAGGGGATCAGGGGATGCCCAGGCATGGACAGTGGGTGGCAGGGGGGGAGAGGAGGGCTGTCTGCTTCCCAGAAGTCCAAGGACACAAATGGGTGAGGGGACTGGGCAGGGTTCTGACCCTGTGGGACCAGAGTGGAGGGCGTAGATGGACCTGAAGTCTCCAGGGACAACAGGGCCCAGGTCTCAGGCTCCTAGTTGGGCCCAGTGGCTCCAGCGTTTCCAAACCCATCCATCCCCAGAGGTTCTTCCCATCTCTCCAGGCTGATGTGTGGGAACTCGAGGAAATAAATCTCCAGTGGGAGACGGAGGGGTGGCCAGGGAAACGGGGCGCTGCAGGAATAAAGACGAGCCAGCACAGCCAGCTCATGTGTAACGGCTTTGTGGAGCTGTCAAGGCCTGGTCTCTGGGAGAGAAGCACAGGGAGGCCAGACAAGGAAGGGGTGACCTGGAGGGACAGATCCAGGGGCTAAAGTCCTGATAAGGCAAGAGAGTGCCGGCCCCCTCTTGCCCTATCAGGACCTCCACTGCCACATAGAGGCCATGATTGACCCTTAGACAAAGGGCTGGTGTCCAATCCCAGCCCCCAGCCCCAGAACTCCAGGGAATGAATGGGCAGAGAGCAGGAATGTGGGACATCTGTGTTCAAGGGAAGGACTCCAGGAGTCTGCTGGGAATGAGGCCTAGTAGGAAATGAGGTGGCCCTTGAGGGTACAGAACAGGTTCATTCTTCGCCAAATTCCCAGCACCTTGCAGGCACTTACAGCTGAGTGAGATAATGCCTGGGTTATGAAATCAAAAAGTTGGAAAGCAGGTCAGAGGTCATCTGGTACAGCCCTTCCTTCCCTTTTTTTTTTTTGTGAGACAAGGTCTCTCTCTGTTGCCCAGGCTGGAGTGGCGCAAACACAGCTCACTGCAGCCTCAACCTACTGGGCTCAAGCAATCCTCCAGCCTCAGCCTCCCAAAGTGCTGGGATTACAAGCATGAGCCACCCCACTCAGCCCTTTCCTTCCTTTTTAATTGATGCATAATAATTGTAAGTATTCATCATGGTCCAACCAACCCTTTCTTGACCCACCTTCCTAGAGAGAGGGTCCTCTTGCTTCAGCGGTCAGGGCCCCAGACCCATGGTCTGGCTCCAGGTACCACCTGCCTCATGCAGGAGTTGGCGTGCCCAGGAAGCTCTGCCTCTGGGCACAGTGACCTCAGTGGGGTGAGGGGAGCTCTCCCCATAGCTGGGCTGCGGCCCAACCCCACCCCCTCAGGCTATGCCAGGGGGTGTTGCCAGGGGCACCCGGGCATCGCCAGTCTAGCCCACTCCTTCATAAAGCCCTCGCATCCCAGGAGCGAGCAGAGCCAGAGCAGGTTGGAGAGGAGACGCATCACCTCCGCTGCTCGCGGCGCGCC |

| **Promoter name** | **Ple32** |
| --- | --- |
| **Description** | Originally derived from human Claudin 5 promoter, sourced from Addgene plasmid #29299: pEMS1503, described in Portales-Casamar et al.^1^ and de Leeuw et al.^2^. |
| **Sequence** | CTTGCCTTCAGAACCTCCCCACACTAGGTGAGCCAGACGCTGGCCTTATCTCATTTACCATCTCAGAGCCATCTGAAGGGGGAGAAGGGAACCGGGCCCCAGGAGGGAGAAAGTCATCAAACCTCCCACATCTGTGACCAGCCTCAGTGCCATACTTTTTCTATGGAGGGCCCTGTCCAATGGAACTGAGCACAGACCAGATAAAAGAACTGGGCACCCAGTGGCCTCAGTCCAGGGCCTGGAGTTCAAACTTTACTGGAAACAAAGGGGCCGAGAGAGACTGGGGAAAGAACTACTAGAAAGGGGCTGGTGCCCCCATGGGGCTGTGGGTTTTGGAGCCGCGTGCCCCCACCTGAGCCTCAGGGGGCCCGGAGTGTCCACACCAGTGGACCTTTCGAGAAATGGCTGGGCCATTGTGCAGAAGAATGCCCGGAAATCCCGCGCCTCCCTCCTCCAGCAAGGATGGGGGCTCTTCCTCCTGGCCAGGAAACTCCAAGTTGGCTTCCGGAGGGTGGCCTGGGGGCTGGGGTGCCAGGGACACCATCGCCACTGGTGGGAGGGCAGGGCACAGCCCCTCCGTGTCCCTTTGTCTCTCCTGTCTGAAGGCCAGAGCAGGCTGCTAGGCCTGGGGCCACCACTGCCCCTGGGTGCTACACCCAGTGTGCTGGGTCACTGGGAACTTCCTGAAGTGGTGTCACCTGAACTGGGCCCCCAAGGATGGGGTGCGGGCAGTACCGCAGGAAGAGGAGCAGCCCCTGTGAAGATTGAGAGGTCTGGGAAGCCCCTGCGGCTTGGGAGAGTGGGGGTCGCCAGGCAGGGGGAAAGCCCCTGTGCCACCGCTTTTTGCCAGAGACTCAGGCTCCAGAGAGGCAGTGAGTGGCATGGGGGGTGAGGCTGGGGCCCTGGGCCTGACCTCCACACGCCTGCCTGGCCTCTCTGTTTGCCATGGGATGAGAGAGACAGTGCTGGGACTCAGAGCGGGGCTGGAGAGTGAGAGTGCGAGAAAGGGCCTGGGTGGGGCTTGGACCCCGGGGCGGGCTTTCTGGAGAGCCCCCCTACGAGGGCCTCTACGGCGGTGACGGGGTGGGGGGCTTCTGCAAACCTTGGTCAGGGAAGTGGAGCTGGCTCGAGTGGAAGAGACCACCCGGCTCAGTCGGGGATGTGGGAGTGGACTGGGTGGTGCAGACTGGGGGTCGAGCGCCTTCTGAAGTGACGGGGCCGGGACGCGCAGGGAGGCGGCCCAAGAAGCGCGCCCTAGGCCAGCCCAGAATGCGCTCGGCCGCGACTAGGACAACGGCGGGTGGGGCTGGGGGCGGCTGCCGGGCGGGGAGCGGTCCCGCGCCCTCAGCTACCCCTCAAGAGCCGTTGTTTCCCTAACTTCAGCTGCCAGAGGCTCTGTGATTGGCTGCGGCACGATGACCCGCGCACGGATTGGCTGCTTCGGGCCGGGGGGCCGGGCCCGGGGGACAGAATCCGCCCCCGAACCTTCAAAGAGGGTACCCCCCGGCAGGAGCTGGCAGACCCAGGAGGTGCGACAGACCCGCGGGGCAAACGGACTGGGGCCAAGAGCCGGGAGCGCGGGCGCAAAGGCACCAGGGCCCGCCCAGGGCGCCGCGCAGCACGGCCTTGGGGGTTCTGCGGGCCTTCGGGTGCGCGTCTCGCCTCTAGCC |

| **Promoter name** | **hSyn1** |
| --- | --- |
| **Description** | Human Synapsin1 promoter |
| **Sequence** | GAGGGCCCTGCGTATGAGTGCAAGTGGGTTTTAGGACCAGGATGAGGCGGGGTGGGGGTGCCTACCTGACGACCGACCCCGACCCACTGGACAAGCACCCAACCCCCATTCCCCAAATTGCGCATCCCCTATCAGAGAGGGGGAGGGGAAACAGGATGCGGCGAGGCGCGTGCGCACTGCCAGCTTCAGCACCGCGGACAGTGCCTTCGCCCCCGCCTGGCGGCGCGCGCCACCGCCGCCTCAGCACTGAAGGCGCGCTGACGTCACTCGCCGGTCCCCCGCAAACTCCCCTTCCCGGCCACCTTGGTCGCGTCCGCGCCGCCGCCGGCCCAGCCGGACCGCACCACGCGAGGCGCGAGATAGGGGGGCACGGGCGCGACCATCTGCGCTGCGGCGCCGGCGACTCAGCGCTGCCTCAGTCTGCGGTGGGCAGCGGAGGAGTCGTGTCGTGCCTGAGAGCGCAGTCGAGA |

| **Promoter name** | **P2-Int** |
| --- | --- |
| **Description** | Transcriptional element 7 (ATF-1/CRE/Sp1/C+TATA Box) as described in US Patent No. 6,346,415 B1, 2002 (<https://www.lens.org/lens/patent/US_6346415_B1>) with the chimeric intron from the CBA promoter. |
| **Sequence** | CTGGAGCCGGTGTCAGGTTGCTCCGGTAACGGTGACGTGCACGCGTGGGCGGAGCCATCACGCAGGTTGCTATATAAGCAGAGCTCGTTTAGTGAACCGTCAGAGGAGTCGCTGCGACGCTGCCTTCGCCCCGTGCCCCGCTCCGCCGCCGCCTCGCGCCGCCCGCCCCGGCTCTGACTGACCGCGTTACTCCCACAGGTGAGCGGGCGGGACGGCCCTTCTCCTCCGGGCTGTAATTAGCGCTTGGTTTAATGACGGCTTGTTTCTTTTCTGTGGCTGCGTGAAAGCCTTGAGGGGCTCCGGGAGCTAGAGCCTCTGCTAACCATGTTCATGCCTTCTTCTTTTTCCTACAGCTCCTGGGCAACGTGCTGGTTATTGTGCTGTCTCATCATTTTGGCAAAGAATTCC |

**1.** Portales-Casamar E, Swanson DJ, Liu L, et al. A regulatory toolbox of MiniPromoters to drive selective expression in the brain. *Proc Natl Acad Sci U S A.* 2010; 107(38):16589-16594.

**2.** de Leeuw CN, Dyka FM, Boye SL, et al. Targeted CNS Delivery Using Human MiniPromoters and Demonstrated Compatibility with Adeno-Associated Viral Vectors. *Mol Ther Methods Clin Dev.* 2014; 1:5.
